# Enzyme-Synthesized Highly Branched Maltodextrins Have Slow Glucose Generation at the Mucosal α-Glucosidase Level and Are Slowly Digestible *In Vivo*

**DOI:** 10.1371/journal.pone.0059745

**Published:** 2013-04-02

**Authors:** Byung-Hoo Lee, Like Yan, Robert J. Phillips, Bradley L. Reuhs, Kyra Jones, David R. Rose, Buford L. Nichols, Roberto Quezada-Calvillo, Sang-Ho Yoo, Bruce R. Hamaker

**Affiliations:** 1 Whistler Center for Carbohydrate Research and Department of Food Science, Purdue University, West Lafayette, Indiana, United States of America; 2 Department of Psychological Science, Purdue University, West Lafayette, Indiana, United States of America; 3 Department of Biology, University of Waterloo, Waterloo, Ontario, Canada; 4 United States Department of Agriculture/Agricultural Research Service Children’s Nutrition Research Center and the Departments Pediatrics, Baylor College of Medicine, Houston, Texas, United States of America; 5 Department of Chemistry, Universidad Autonoma de San Luis Potosi, San Luis Potosi, Mexico; 6 Department of Food Science & Technology and Carbohydrate Bioproduct Research Center, Sejong University, Seoul, Korea; Wageningen University, The Netherlands

## Abstract

For digestion of starch in humans, α-amylase first hydrolyzes starch molecules to produce α-limit dextrins, followed by complete hydrolysis to glucose by the mucosal α-glucosidases in the small intestine. It is known that α-1,6 linkages in starch are hydrolyzed at a lower rate than are α-1,4 linkages. Here, to create designed slowly digestible carbohydrates, the structure of waxy corn starch (WCS) was modified using a known branching enzyme alone (BE) and an in combination with β-amylase (BA) to increase further the α-1,6 branching ratio. The digestibility of the enzymatically synthesized products was investigated using α-amylase and four recombinant mammalian mucosal α-glucosidases. Enzyme-modified products (BE-WCS and BEBA-WCS) had increased percentage of α-1,6 linkages (WCS: 5.3%, BE-WCS: 7.1%, and BEBA-WCS: 12.9%), decreased weight-average molecular weight (WCS: 1.73×10^8^ Da, BE-WCS: 2.76×10^5^ Da, and BEBA-WCS 1.62×10^5 ^Da), and changes in linear chain distributions (WCS: 21.6, BE-WCS: 16.9, BEBA-WCS: 12.2 DP_w_). Hydrolysis by human pancreatic α-amylase resulted in an increase in the amount of branched α-limit dextrin from 26.8% (WCS) to 56.8% (BEBA-WCS). The α-amylolyzed samples were hydrolyzed by the individual α-glucosidases (100 U) and glucogenesis decreased with all as the branching ratio increased. This is the first report showing that hydrolysis rate of the mammalian mucosal α-glucosidases is limited by the amount of branched α-limit dextrin. When enzyme-treated materials were gavaged to rats, the level of postprandial blood glucose at 60 min from BEBA-WCS was significantly higher than for WCS or BE-WCS. Thus, highly branched glucan structures modified by BE and BA had a comparably slow digesting property both *in vitro* and *in vivo*. Such highly branched α-glucans show promise as a food ingredient to control postprandial glucose levels and to attain extended glucose release.

## Introduction

Starch is classified into three nutritional types: rapidly digestible starch (RDS), slowly digestible starch (SDS), and resistant starch (RS) [1]. SDS has drawn recent interest, because foods containing SDS are considered to have a low-glycemic index (GI) with extended glucose release [2], [3], and may be particularly important for individuals having diabetes and pre-diabetes [4], [5]. Also, glucose release from glycemic carbohydrates (including starch and maltose) in the ileum stimulates the “ileal break” which is known to decrease gastric emptying that is related to food intake control and satiety levels [6], [7]. SDS-containing ingredients or foods are difficult to achieve, and are often transient in nature due to processing and storage conditions. Some recognized ways to develop SDS are with annealing/heat-moisture treatment [8], recrystallization [9], and enzyme treatment [10]; though the first two of these materials are subject to loss during cooking. Most desirable is to have the slow glucose release property structurally inherent to the material so that it is retained through processing or home cooking. Enzymatic modification of starch offers this possibility.

**Figure 1:**
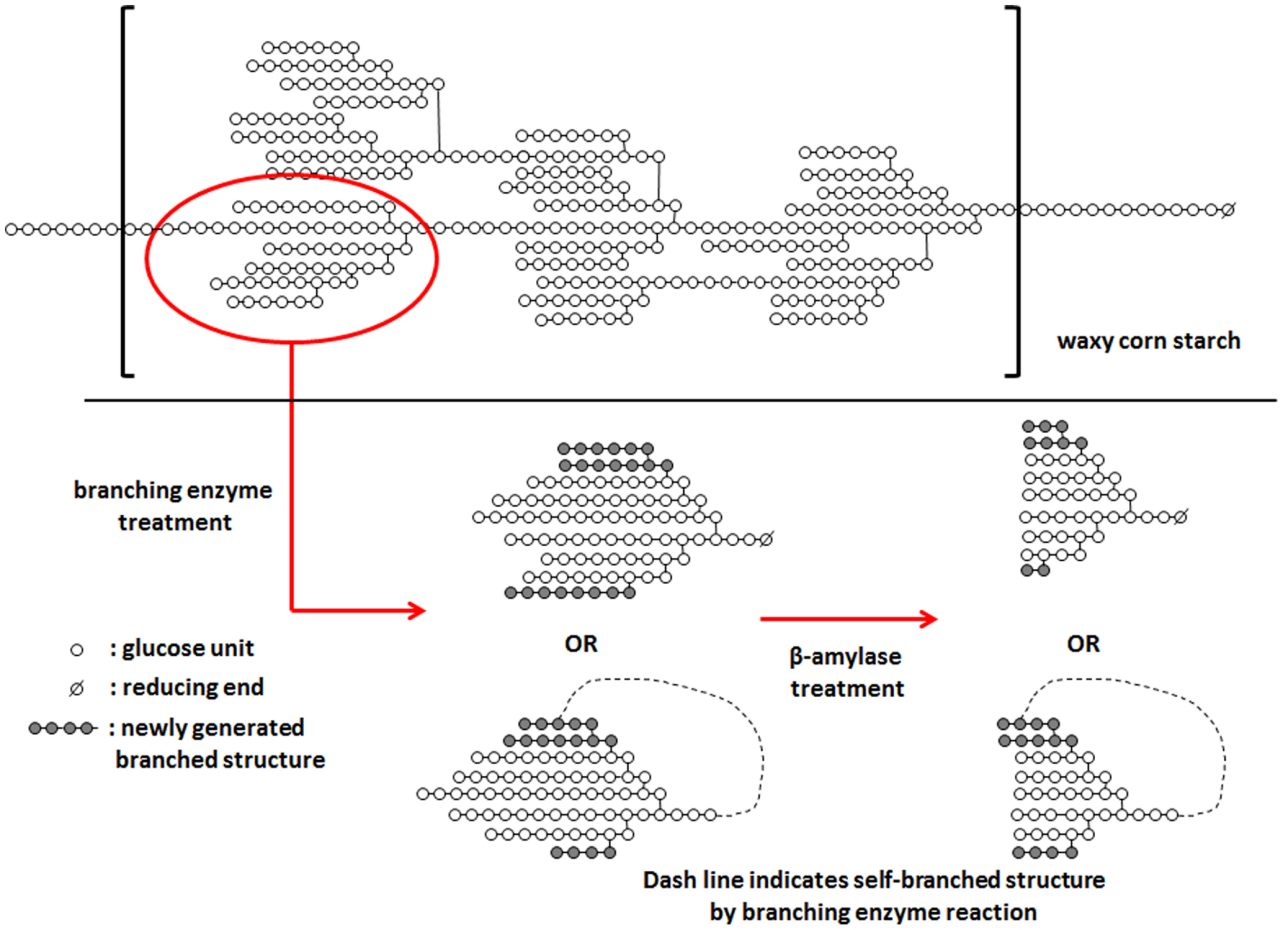
Schematic diagram for action patterns of branching enzyme and β-amylase to make highly branched maltodextrins. Branching enzyme was used first to hydrolyze α-1,4 linkages on the waxy corn starch molecule, and to generate new α-1,6 linkages. β-Amylase further was used to hydrolyze α-1,4 linkages to increase the α-1,6 linkage ratio.

A strategy to produce enzyme-modified starch-based materials for slow glucose release is to increase branch density, which results in slower *in vitro* digestion rate due to the lower hydrolysis rate of α-1,6 linkages compared to α-1,4 linkages [3]. In this regard, branching enzyme (BE, EC. 2.4.1.18) catalyzes the hydrolysis of α-1,4 linked linear chains followed by glycosyltransferase action to create a new α-1,6 linked branch chains [11]–[13], often in a cyclized form [10], and produces highly branched maltodextrins which have comparably slower digestible properties [13], [14]. β-Amylase (BA, EC. 3.2.1.2), which hydrolyzes external α-1,4 linkages, has been applied to partially shorten external chain length to increase starch branch density ratio [12], [15]. In the present study, we synthesized highly branched structures from WCS using BE treatment as above, and by combining BE and BA treatments. These products are proposed to have the structures shown in Figure 1 (adapted from reference [10]).

For digestion of starch to glucose, different types of α-glycosidic hydrolases are involved. Initially, the salivary and pancreatic α-amylases break down the starch structure to α-limit dextrins [16], which we define here to consist of linear (mainly maltose and maltotriose) and branched maltooligosaccharides. The α-limit dextrins are then digested to glucose by action of the maltase-glucoamylase (MGAM) and sucrase-isomaltase (SI) complexes in the small intestine [17], [18]. Both MGAM and SI complexes are anchored to the brush-border membrane [19] and each contains two catalytic enzymes, denoted using current terminology as N-terminal (ntMGAM and ntSI) and C-terminal (ctMGAM and ctSI) α-glucosidases [20]–[22]. The mucosal α-glucosidases belong to the glycosyl hydrolase Family 31 (GH31) based on their hydrolytic properties and sequence identity (40–60%) [23]–[25]. Although each mucosal α-glucosidase has somewhat different hydrolytic properties on the α-glycosidic linkages, all have activity on the α-1,4 linkage which is the major linkage in the backbone of the starch structure [17], [21]. Additionally, ntSI and ntMGAM have debranching activity on branched α-limit dextrins with the former having the dominant α-1,6 linkage hydrolysis activity [26], [27].

With previous studies having shown that highly branched α-glucans reduce rate of α-amylolysis [13], [28], the hypothesis tested in this study was whether the α-limit dextrin products from these structures, and particularly the BEBA material newly designed in this study, decelerate glucogenesis at the mucosal α-glucosidase level. This would be primarily due to the higher *K*_m_ values of mucosal ntSI and ntMGAM for the α-1,6 linkage, than for the α-1,4 linkage each of the four α-glucosidases [27], which would slow glucose generation. Our findings provide further information for the design of carbohydrates that have slowly digestible properties at the mucosal α-glucosidase level.

## Materials and Methods

### Ethics Statement

The rat study was undertaken with approval from the Purdue Animal Care and Use Committee.

### Materials

Waxy corn starch (Tate and Lyle, Inc. Decatur, IL) was used as a substrate for enzymatic modification. Branching enzyme (BE) from *Rhodothermus obamensis* (Branchzyme®) was a gift from Novozymes North America, Inc. (Franklinton, NC) and β-amylase from barley extract (Optimalt® BBA) was gift from Danisco US Inc., Genencor Division (Cedar Rapids, IA). The glucose assay kit was purchased from Megazyme (Wicklow, Ireland). All other chemicals were purchased from Sigma-Aldrich Co. (St. Louis, MO).

### Preparation of Enzyme-Modified Starch Products

Waxy corn starch (WCS) was suspended in 50 mM sodium acetate buffer (pH 6.5, 25% w/v, 100 mL) and was boiled to a paste. The suspension was incubated with stirring with branching enzyme (BE, 500 U/g dry weight of starch_,_ Novozymes unit) at 65°C for 24 h to produce BE-treated WCS (BE-WCS). After 24 h, BE was inactivated with in a boiling water bath for 10 min. To further reduce the proportion of the α-1,4 linkages, BE-WCS solution (95 mL) was mixed with 1 M sodium acetate buffer (5 mL, pH 5.0) and stirred for 10 min. The mixture was incubated with β-amylase (BA, 0.64% dry weight of starch) at 55°C for 24 h to produce BEBA-WCS. The BA was inactivated in a boiling water bath [15]. Then, both BE- and BEBA-WCS were dialyzed (MWCO: 3,500) in de-ionized water to remove the released oligosaccharides (mainly maltose) from the β-amylase reaction, as well as salt ions from the buffer solution. The samples were lyophilized, ground (Lab Grinder A10S1, Janke & Kunkel IKA Labortechnik, Germany), and passed through a 120 mesh sieve.

### Analysis of Debranched Linear Chain Length Distribution by HPAEC

Enzyme-treated starch solutions containing WCS (900 µL, 1%, w/v) were combined with 100 mM sodium acetate buffer (100 µL, pH 5.0). The mixture was incubated with pullulanase (0.72 units, Megazyme, Wicklow, Ireland) and isoamylase (0.1 units, Megazyme, Wicklow, Ireland) at 40°C for 48 h, resulting in the hydrolysis of the α-1,6 linkages [29]. Debranched linear chain length distributions of enzyme-treated starch were measured on a high-performance anion-exchange chromatograph (HPAEC) fitted with an electrochemical detector (ED40, Dionex, Sunnyvale, CA). Filtered samples (with 0.22 µm, 25 µL) were injected into a CarboPac PA-100 pellicular anion-exchange column (Dionex, Sunnyvale, CA) that was pre-equilibrated in eluent A (150 mM NaOH) at 1.0 mL/min. Chromatographic separation of the linear oligosaccharides from the sample was achieved by gradient elution from 100% eluent A to 100% eluent B (600 mM sodium acetate in 150 mM NaOH) [13]. The chain length distribution was characterized as a percentage of the total peak area [30].

### Analysis of Molecular Size Distributions by High Performance Size-Exclusion Chromatography (HPSEC) with MALS-RI

Starch samples were dissolved in de-ionized water (10 mg/mL, w/v), and boiled for 20 min with stirring. Weight-average molecular weight (M_w_) and molecular size distributions of enzyme-modified WCS were determined using multi-angle laser light scattering (MALS, Dawn Heleos-II, Wyatt Tech. Corp., Santa Barbara, CA) and refractive index (RI, Optilab rEX, Wyatt Tech. Corp., Santa Barbara, CA) detectors at 35°C, respectively. Injected samples (200 µL, passed through a 5 µm nylon filter) were separated on Sephacryl™ S-500 HR gel filtration media (GE Healthcare, Piscataway, NJ). Mobile phase was purified water (18.2 mΩ) with 0.02% sodium azide at a flow rate of 1.3 mL/min [31]. The collected data from MALS and RI detectors were analyzed using a Berry plot for curve fitting with Astra software version V (Wyatt Tech. Corp., Santa Barbara, CA), and a *dn/dc* value of 0.146 mL/g was applied for M_w_ calculation [32], [33].

### Analysis of Linkage Ratio by Proton Nuclear Magnetic Resonance (^1^H NMR) Spectroscopy

The relative abundance of α-1,4 and α-1,6 linkages in the enzyme-modified WCS samples were determined by proton nuclear magnetic resonance (^1^H NMR) spectroscopy (Varian Unity Inova 300 MHz, Varian INC., Palo Alto, CA) [34]. Freeze-dried enzyme-modified starch samples (20 mg/mL) were first dissolved in deuterium oxide (D_2_O), and then boiled with stirring for 30 min. The samples were freeze-dried again, and samples, which were re-dissolved in D_2_O (20 mg/mL), were analyzed by ^1^H NMR analysis. ^1^H NMR spectra were collected at 80°C.

### Preparation of the Recombinant MGAM and SI α-Glucosidases

Each mammalian mucosal α-glucosidase was expressed via a baculovirus system through different host insect cells [21]. The recombinant baculovirus for ctSI and ctMGAM, named as pAcGP67 His-ctSI and pAcGP67 His-ctMGAM, respectively, were transfected into the Sf9 insect cell. The ntMGAM fragment was ligated into a *Drosophila* pMT-BiP-V5-His vector, and ntSI was cloned into a *Drosophila* pMT-TEVA expression vector [35]. These N-terminal recombinant vectors were transfected to *Drosophila* S2 cells to express recombinant protein. The released soluble proteins from recombinant insect cells were purified with a nickel-nitrilotriacetic acid (Ni-NTA) affinity column chromatography (Qiagen, Hilden, Germany) by changing of imidazole concentration (0–250 mM). The purified recombinant α-glucosidases were concentrated by centrifugal devices (Microcon YM-30, MWCO 30,000; Millipore, CA) and the protein concentration was determined by the Bradford method using a bovine serum albumin (BSA) standard [36].

### Hydrolysis Properties by Human Pancreatic α-Amylase and Mammalian Mucosal α-Glucosidases

Hydrolysis property of the enzyme-modified WCS’s by human pancreatic α-amylase was investigated. Enzyme-modified starches were solubilized in 10 mM PBS buffer (pH 6.9, 2 mg/mL, w/v) and reacted with human pancreatic α-amylase (500 U, Meridian Life Science, Inc., Saco, Maine) at 37°C for 24 h. The solution was heat-treated for 5 min in a boiling water bath to inactivate the enzyme. The α-amylolyzed sample was passed through a 0.45 µm syringe filter and injected into an HPSEC system equipped with Superdex 200 prep grade gel and Superdex 30 Prep grade gel (GE Healthcare, Piscataway, NJ) columns. The mobile phase was purified water (18.2 mΩ) with 0.02% sodium azide at a flow rate of 0.4 mL/min [37].

The enzyme-modified starch products (including WCS) were hydrolyzed by four different recombinant mucosal α-glucosidases. Before hydrolysis, the samples were pre-incubated with human pancreatic α-amylase (500 U) at 37°C for 24 h to produce α-limit dextrins (fully α-amylolyzed starch). Each mucosal α-glucosidase (500 U, one unit (U) enzyme activity arbitrarily defined as 1 µg of glucose released from 1% maltose per 10 min at 37°C) was reacted with 1% (w/v) of α-amylase-treated substrates in 10 mM PBS buffer (pH 6.8) at 37°C. The amount of released glucose was analyzed by the glucose oxidase/peroxidase (GOPOD) method [38].

### *In Vivo* Digestibility Properties using the Rat Model

Sprague-Dawley male rats (6 rats, 8 weeks old, 443.2 g ±23.4) were used. Each animal received all treatments on different test days. A randomized design was used. They were habituated for a few days prior to the test day. The three enzyme-modified starches were gavaged via the mouth as the means of administration of treatments. Glucose was used for the digestible high glycemic control instead WCS due to the high viscosity of the latter, which would have been a confounding factor in the study. Baseline blood glucose was taken from the tail at zero time, and 15, 30, 60 and 120 min following ingestion of the samples. Collected blood from the rat tail was analyzed using a blood glucose monitoring system (Bayer Contour Meter, Bayer HealthCare LLC, Tarrytown, NY). Area under the curve (AUC) was calculated by the trapezoidal rule [39], and collected data were analyzed using SAS software (version 9.2, SAS institute, Cary, NC). Differences between least square means at each time point were evaluated by Tukey’s tests and statistical significance was considered at *P*<0.05.

## Results and Discussion

Until recently [22], [40], it was considered that the α-glucosidases hydrolyze starch almost immediately to glucose irrespective of α-limit dextrin structure. The linear structures are comprised of maltose, maltotriose, and a minor amount of maltotetraose, and the branched structures can be a wide array of small α-glucans with one or more α-1,6 branch points. The findings of the current study show that digestion of the α-limit dextrin fraction occurs by all four α-glucosidases, and that the higher degree of branching in the enzyme-synthesized materials retards its digestion by all the subunits. Because α-amylase itself has no debranching activity, it seems plausible that the rate-limiting step in digestion of the highly branched materials is at the mucosal α-glucosidase level, and translates to its slower digestion.

### Debranched Linear Chain Length Distribution of Enzyme-Modified Starch by HPAEC

**Figure 2:**
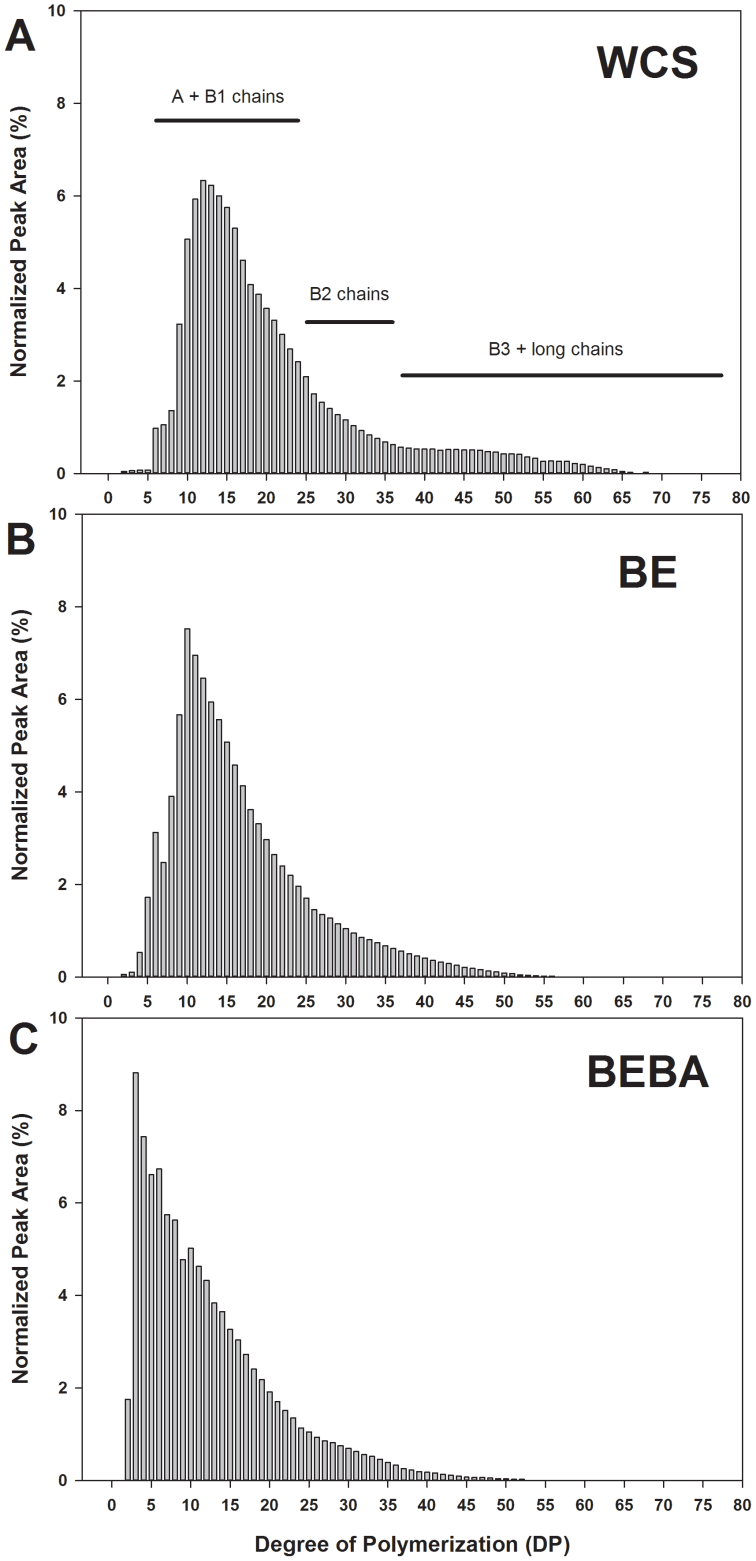
Debranched linear chain length distribution of enzyme-modified starches determined by high performance anion exchange chromatography (HPAEC): A, waxy corn starch; B, branching enzyme-treated WCS (BE-WCS); and C, β-amylase-treated BE (BEBA-WCS).

Overall debranched linear chain length distribution was shortened considerably by enzyme modification with BE and BA on waxy corn starch (WCS) (Figure 2). The data from the debranched linear chain distribution of native WCS showed the largest fraction at DP 12, corresponding to the A and B1chains as reported by Hanashiro, Abe et al. (1996) (Figure 2A) [30]. Side chain distribution of BE-treated WCS (BE-WCS) showed a considerable decrease in the proportion of B3 and longer chains (DP>37) with little decrease in external short chain length (peak at DP 10) (Figure 2B). Thus, rearrangement of side chains were produced by the BE reaction on α-1,4 linkages and transfer the cleaved moieties to generate new α-1,6 linkages [41]. BA-treated BE-WCS (BEBA-WCS) reduced the DP of the main peak to 3 due to the action of β-amylase (Figure 2C). However, the distribution of B2 and longer chains on BE-WCS was not noticeably decreased by β-amylolysis, because these represent branched portions of the molecule which β-amylase does not hydrolyze. Weight and number-average degree of polymerization (DP_w_ and DP_n_) of WCS were considerably decreased from 21.6 to 12.2 and from 15.4 to 7.4, respectively, after BE and BEBA treatments (Table 1).

**Table 1:** Change of degree of polymerization (DP) of the enzyme-modified starch.

	WCS	BE-WCS	BEBA-WCS
DP*_w_*	21.6	16.9	12.2
DP*_n_*	15.4	13.0	7.4

WCS: waxy corn starch.

BE-WCS: branching enzyme-treated WCS.

BEBA-WCS: β-amylase treated BE-WCS.

DP_w_: The weight-average degree of polymerization.

DP_n_: The number-average degree of polymerization.

### Molecular Size Distribution of Enzyme-treated Starch Products

**Figure 3:**
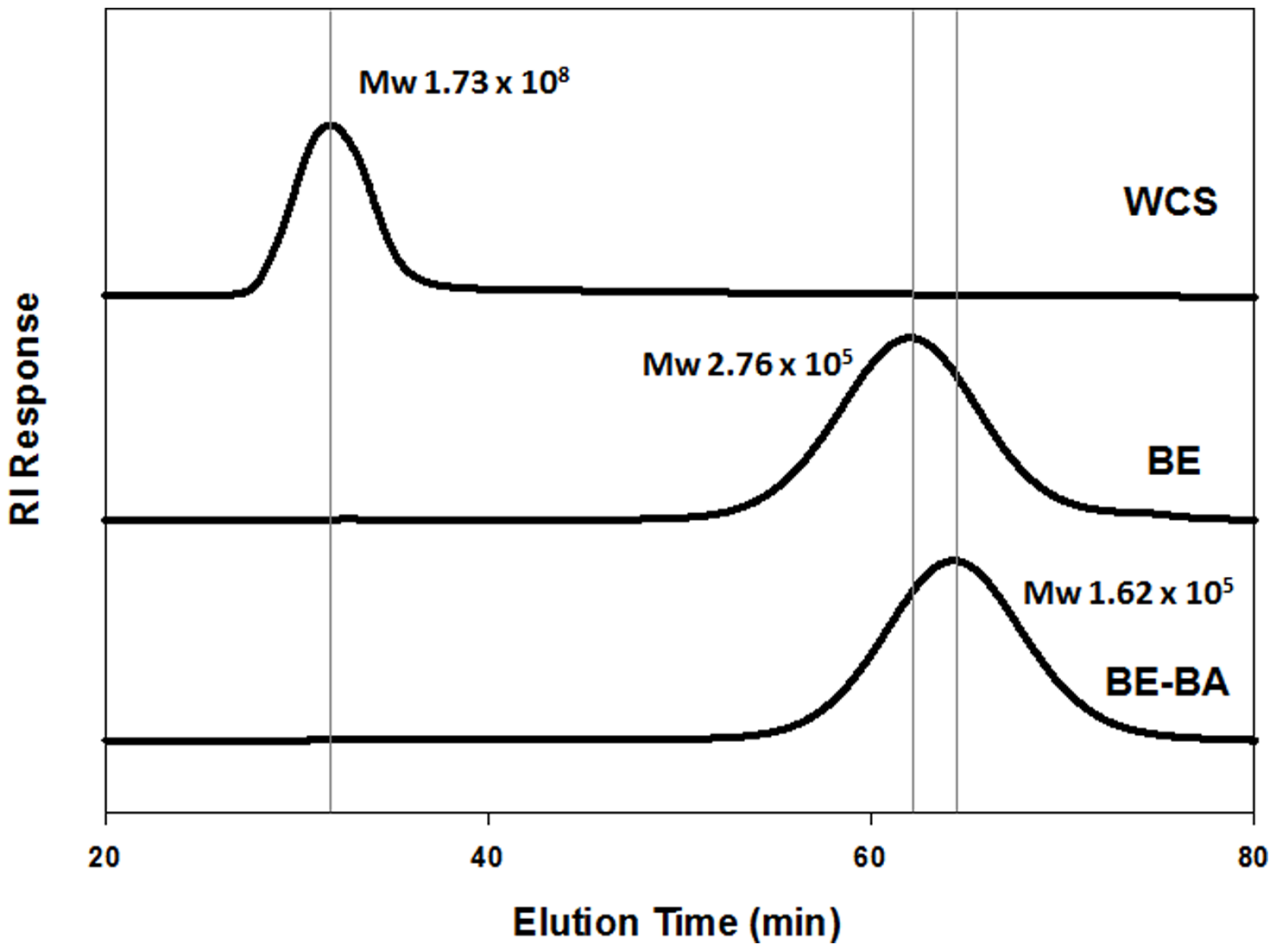
Molecular size distribution and weight-average molecular weight of enzyme-treated waxy corn starches by HPSEC-MALS-RI. Branching enzyme treatment decreased the molecular size of waxy corn starch due to its hydrolytic properties on α-1,4 linkages. The molecular size was further decreased after β-amylase treatment. WCS, waxy corn starch; BE, branching enzyme-treated WCS; and BE-BA, β-amylase-treated BE. M_w_, weight-average molecular weight (g/mole).

Figure 3 shows data for the molecular size distributions and weight-average molecular weight (M_w_) of enzyme-treated starch products as measured by multi-angle laser light scattering (MALS) and refractive index (RI) detection. The M_w_ of WCS was 1.7×10^8 ^Da, while that of BE-WCS was decreased to 2.76×10^5^ Da. Amylopectin consists of multiple clusters connected by B2 and longer chains (DP 25<) [30]. Based on enzymatic properties of BE, hydrolysis of α-1,4 linkages between clusters leads to decrease in M_w_ in the waxy corn starch (WCS) [42]. This decrease in M_w_ increased the number of short chains (A chains and B1) by α-glycosyl transferring activity of BE [28]. β-Amylase treatment (BEBA-WCS) led to a further decrease in M_w_ to 1.62×10^5 ^Da compared to BE-WCS due to hydrolysis of the external linear chains. Cave et al. [43] noted that starch can be degraded by shear scission as flow rate is increased during HPSEC analysis, and suggests the use of slow flow rates. Our systems have low back pressures using intermediate pressure columns at the flow rates indicated, with negligible scission expected. Le et al. [28], in chromatographing hydrolyzed starch molecules of a similar molecular size range, showed results comparable to ours.

### Linkage Ratios by ^1^H NMR Spectroscopy

^1^H NMR was applied to analyze the ratio of α-1,6 linkages to α-1,4 linkages in the samples from the both BE-derived transferring reaction and β-amylolysis. In this research, maltose and panose were used as standards for determining the chemical shifts of the resonances from the H-1 positions of α-1,4 and α-1,6 linkages. The percentages of the linkages were calculated to measure the branching ratio of samples.

The relative integration value for the resonance for the 1,6 linkages in WCS was 5.3%, while it was 7.1% for BE-WCS and for 12.9% in BEBA-WCS (Table 2). Thus, the branching ratio was significantly increased (*P*<0.05) by enzyme treatments. This data combined with the debranched linear chain length distributions suggests that the enzymatically synthesized-highly branched products were good candidates for slowly digestible carbohydrates.

**Table 2:** Relative abundance (%) of α-1,4 and α-1,6 linkages in the enzyme-modified starches.

Sample	α-1,4 linkages (%)	α-1,6 linkages (%)	Ratio of α-1,4 to α-1,6
Maltose	100	0	–
Panose	50	50	1
WCS	94.7	5.3	18.0^a^ ±2.0
BE-WCS	92.9	7.1	11.3^b^ ±3.0
BEBA-WCS	87.1	12.9	6.8^c^ ±0.1

Percentage were determined by calculating the area ratio from ^1^H NMR.

Significant differences are expressed as different superscript letters (*P*<0.05).

±:standard deviation.

WCS: waxy corn starch.

BE-WCS: branching enzyme-treated WCS.

BEBA-WCS: β-amylase treated BE-WCS.

### Quantification of α-Limit Dextrin Amount

**Figure 4:**
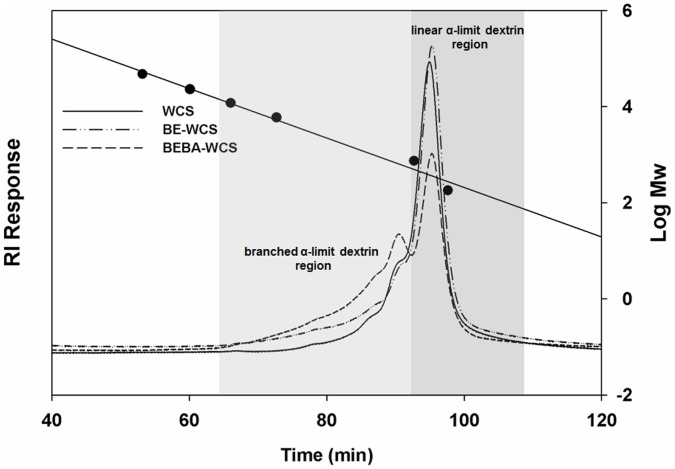
Molecular size distribution of enzyme-treated starches by HPSEC-RI after human pancreatic α-amylase treatment. The two different regions stand for branched and linear α-limit dextrins, respectively. WCS, waxy corn starch; BE-WCS, branching enzyme-treated WCS; BEBA-WCS, β-amylase-treated BE-WCS.

Change in amount of branched α-limit dextrin, products of enzyme-modified WCS by BE and the combination of BE and BA, was evaluated after their complete hydrolysis using human pancreatic α-amylase. Figure 4 shows size-exclusion chromatographic tracings with two different regions, linear and branched α-limit dextrins, after α-amylolysis of the WCS-based samples, as first shown by Jones, Brown et al (1983) [16]. The proportion of branched α-limit dextrin substantially increased (WCS: 26.8%, BE-WCS: 30.4%, and BEBA-WCS: 56.8%) with the BE and BEBA treatments (Table 3), thus providing appropriate structures for testing of the hypothesis that highly branched structures affect the hydrolysis rate at the mucosal α-glucosidase level.

**Table 3:** The amount of peak area (%) between branched and linear maltooligosaccharide regions.

Sample	Area for branched (α-1,6 linked) oligosaccharides	Area for linear (α-1,4 linked) maltooligosaccharides
WCS	26.8	73.2
BE-WCS	30.4	69.6
BEBA-WCS	56.8	43.2

WCS: waxy corn starch.

BE-WCS: branching enzyme-treated WCS.

BEBA-WCS: β-amylase treated BE-WCS.

### Hydrolysis by Individual Recombinant Mucosal α-Glucosidases

**Figure 5:**
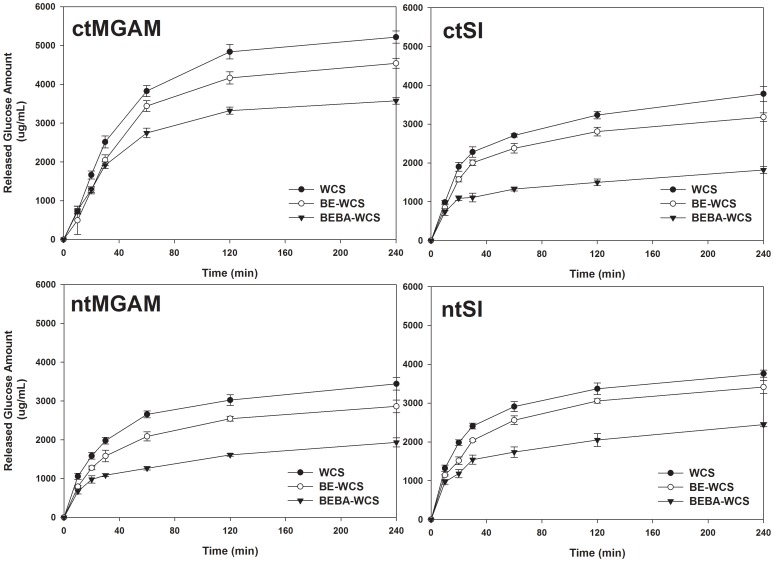
Hydrolysis properties of enzyme-modified maltodextrins (1%, w/v) using the four mucosal α-glucosidases (500 U). As the amount of α-1,6 linkages were increased, the released glucose amounts decreased by the individual mucosal α-glucosidase reactions. WCS, waxy corn starch; BE-WCS, branching enzyme-treated WCS; and BEBA-WCS, β-amylase-treated BE-WCS. ctMGAM, C-terminal maltase-glucoamylase; ntMGAM, N-terminal maltase-glucoamylase; ctSI, C-terminal sucrase-isomaltase; and ntSI, N-terminal sucrase-isomaltase.

The highly branched α-limit dextrins were expected to release glucose slowly by mucosal α-glucosidases due to lesser amount of linear maltooligosaccharides as well as the relatively slow hydrolysis of the α-1,6 linkage. Notably, all four mucosal α-glucosidases (ctMGAM, ntMGAM, ctSI, ntSI) showed decreased hydrolysis rate, as measured by glucose release, with increase in branch points of α-limit dextrins (Figure 5). This provides clear evidence that hydrolysis rate of the mucosal α-glucosidases is limited by the amount of branched structures.

In the case of C-terminal α-glucosidases which only hydrolyze α-1,4 linkages, linear maltooligosaccharides from α-amylolysis (mainly maltose and maltotriose) are quickly hydrolyzed to glucose. For the branched α-limit dextrins, only the available linear α-1,4 linkages from the non-reducing end until the branch point can be hydrolyzed to glucose by ctMGAM and ctSI. This explains the lower amount of hydrolysis by the C-terminal α-glucosidases with increasing amount of branched α-limit dextrins (Figure 5). Interestingly, the amount of glucose released from the three α-limit dextrins by ctMGAM was higher compared to that of the other mucosal α-glucosidases with same enzyme amount added (500 U). The result can be explained by high α-1,4 hydrolytic rate of ctMGAM on long maltooligosaccharide and α-limit dextrins [22], though single ctMGAM treatment cannot fully hydrolyze the substrate molecules due to no debranching activity.

Hydrolysis of enzyme-modified WCS by the N-terminal α-glucosidases also decreased as the branching ratio of the structures increased (Figure 5). In case of ntMGAM, though this enzyme has small debranching activity, its hydrolytic rate (*k*_cat_/*K*_m_: 0.06 s^−1^mM^−1^) was approximately 150 times lower than isomaltase activity for ntSI (*k*_cat_/*K*_m_: 9 s^−1^mM^−1^), and 400 times lower than maltase activity for ntMGAM (*k*_cat_/*K*_m_: 26 s^−1^mM^−1^) [27]. Thus the α-1,6 linkage debranching property of ntMGAM is negligible during normal starch digestion. Although ntSI preferably hydrolyzes the α-1,6 linkage, this hydrolysis is slower than for ntSI hydrolysis of the α-1,4 linkage (*k*_cat_/*K*_m_: 19 s^−1^mM^−1^). The hydrolytic rate of ntSI is decelerated as it encounters α-1,6 linked glucose as the enzyme hydrolyzes from the non-reducing ends of the branched α-limit dextrins. Hence, the enzymatically synthesized highly branched maltodextrins were slowly hydrolyzed by ntMGAM due to lower percentage of linear maltooligosaccharides and by ntSI due to this and its slower hydrolyzing property for α-1,6 linkages.

As the available amount of fast-digesting linear maltooligosaccharides decreased, overall digestion concomitantly decreased. Also, α-1,6 linkages in the branched α-limit dextrins were shown to be slowly hydrolyzed by ntSI. Thus, at *in vivo* level, it was expected that the enzymatically synthesized highly branched maltodextrin would be digested more slowly by the combined action of all mucosal α-glucosidases.

### *In Vivo* Digestibility Properties using the Rat Model

**Figure 6:**
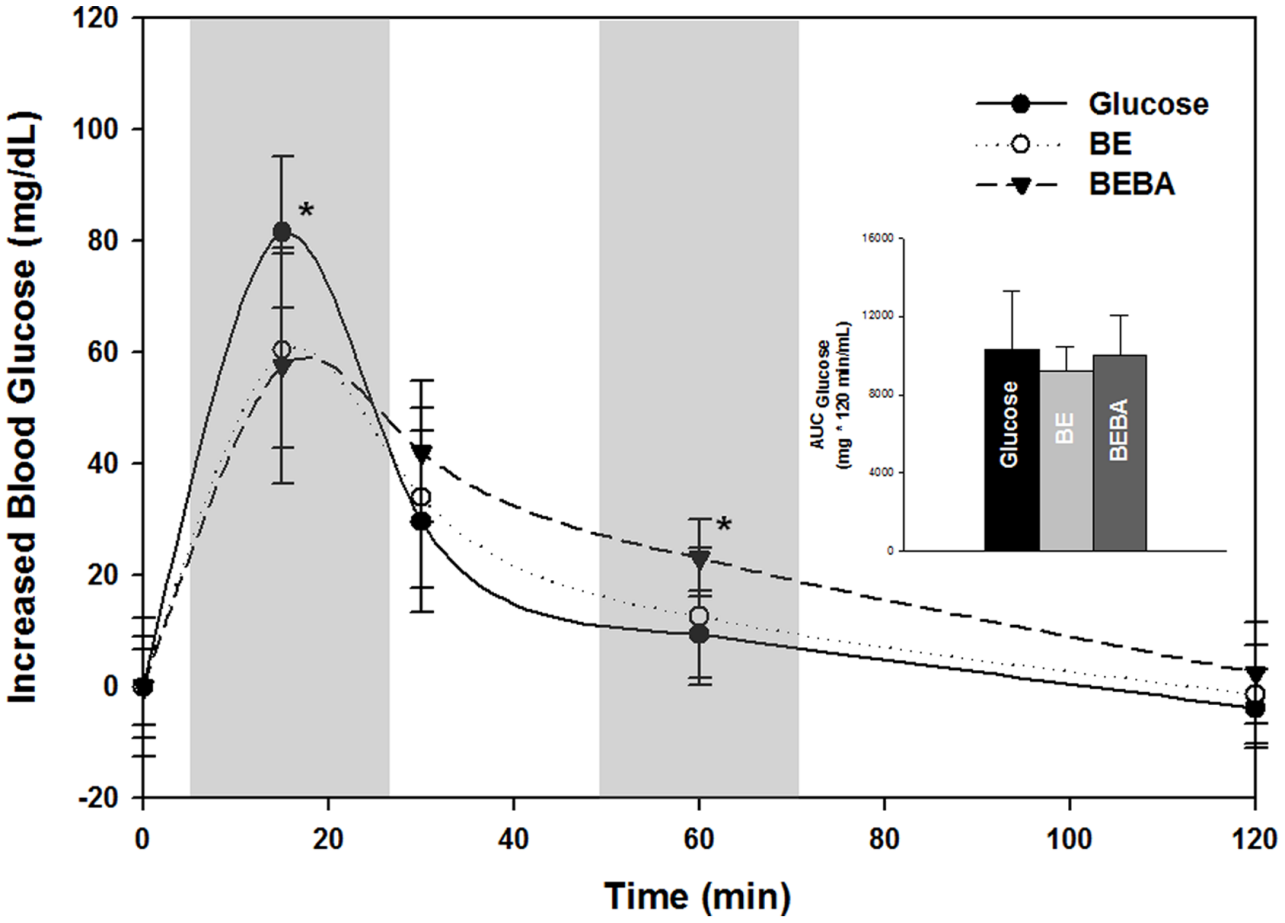
Glycemic response of enzyme-modified maltodextrins using a rat model. Decreased blood glucose peak level (mg/dL) is observed in rats with BE and BEBA enzyme-modified maltodextrins, and increased blood glucose level was found at 60 min for the BEBA maltodextrin. The inset graph indicates no significant difference in area under the curve (AUC). BE, branching enzyme-treated WCS; BEBA, β-amylase-treated BE. Asterick (*) indicates significant difference at *P*<0.05.

Figure 6 presents *in vivo* postprandial glycemic response profiles using the enzyme-modified starches with different branching ratios. Area under the curve (AUC) of blood glucose profiles showed no significant differences (*P*<0.05) among different samples, implying that the amount of released glucose was the same. Postprandial blood glucose level at 15 min was significantly higher for the glucose control (*P*<0.05) compared to the BE- and BEBA-WCS, though the enzyme-modified samples still had moderately high blood glucose levels presumably due to ease of digestion of the linear-contained maltooligosaccharides. Notable was the finding that BEBA-WCS had a significantly higher blood glucose response at 60 min indicating that the branched structures retard digestion rate. The present work extends a previously reported short term glycemia difference in mice resulting from ingestion of highly branched cluster cyclodextrins (HBCD) [14], which are produce by branching enzyme from *Bacillus stearothermophilus* [10]. Those authors showed that HBCD had a lower glycemic response at 10 min, while blood glucose decreased to the same level as the control at 30 min. Likewise, in the present study using rats, BE-WCS statistically had the same blood glucose level as the glucose control at 60 min. However, the more highly branched BEBA-WCS showed an extended glucose release that was higher than BE-WCS and the glucose control at 60 min. This is presumed to be due to the higher amount of branched α-limit dextrin (56.8%) in the BEBA-WCS sample. Therefore, *in vivo* results support our hypothesis that carbohydrate digestion is affected by proportion of α-1,6 linkages leading to higher amounts of branched α-limit dextrin.

### Conclusions

Increased branch density of original substrates, which led to increase in the amount of branched α-limit dextrin, clearly shows a reduction in hydrolysis by four mucosal α-glucosidases. Thus, amount of α-1,6 linked branches in starch and starch products can be considered as a way to control glucogenesis *in vivo.* This represents an opportunity to design specific α-glucan-based substrates to control glucogenesis by the action of mucosal α-glucosidases resulting in moderation of glycemic profiles and slowly digestible properties.
